# Purple-Grain Wheat Regulation of Blood Lipids and Blood Glucose in Diet-Induced Hyperlipidemic Mice and Type 2 Diabetic Mice

**DOI:** 10.3390/nu17081310

**Published:** 2025-04-09

**Authors:** Dong Hu, Shasha Cheng, Xiaoyan Wei, Chaoxin Man

**Affiliations:** 1 Key Laboratory of Dairy Science, Ministry of Education, College of Food Science, Northeast Agricultural University, Harbin 150030, China; donghu1983@163.com (D.H.);; 2 Institute of Agro-Resources and Environment/Hebei Fertilizer Technology Innovation Center, Hebei Academy of Agriculture and Forestry Sciences, Shijiazhuang 050000, China; weixiaoyanyu@163.com

**Keywords:** purple-grain wheat, glucolipid level, hyperlipidemia, type 2 diabetes

## Abstract

**Background/Objectives:** Disorders of glucose and lipid metabolism can easily lead to metabolic diseases such as hyperlipidemia and diabetes mellitus, with multiple complications. This study evaluated the regulatory effect of purple-grain wheat on glycolipid metabolism. **Methods:** In this study, we established a hyperlipidemic mouse model by means of a high-fat diet and a type 2 diabetic mouse model using a high-fat and high-sugar diet combined with streptozotocin, and the mice were intervened with 15 g/(kg·d), 7.5 g/(kg·d), and 3.75 g/(kg·d) doses of purple-grain wheat paste (PWP) for 4 and 5 weeks, respectively. **Results:** The results revealed that PWP reversed the increase in body weight; increased serum high-density lipoprotein cholesterol; and decreased serum total cholesterol, triglycerides, and low-density lipoproteins. In addition, PWP reversed the decrease in body weight and alleviated the sustained increase in blood glucose in type 2 diabetic mice. **Conclusions:** PWP shows a significant ability to regulate glycolipid levels, which is related to its functional composition and its ability to act as a prebiotic. In conclusion, PWP can be considered a potential functional food for lowering blood glucose and blood lipids.

## 1. Introduction

With the rapid development of the global economy, people’s diets and lifestyles have changed dramatically. The incidence of chronic diseases such as obesity, type 2 diabetes mellitus, and hyperlipidemia, which are commonly characterized by disorders of glucolipid metabolism, has risen sharply creating a serious test for public health in modern society [1]. Genetics and a high-energy diet are recognized as the two major causes of glycolipid metabolism disorders in the human body. Due to the close correlation between dietary structure and human health, in recent years, the research on a healthy diet and its functional factors and active mechanisms has become a key direction in the field of modern food science research, in which the regulatory effect of dietary fiber on human glycolipid metabolism is of particular interest. The biological activity of dietary fiber in the prevention and treatment of diseases related to disorders of glycolipid metabolism has been confirmed by numerous studies [2,3,4]. Gut microbes play a very important role in glycolipid metabolism in the body through host–diet interactions. Dietary fiber reduces the amount of bile acids entering the hepatic and intestinal circulation by adsorbing bile acids, thereby promoting the conversion of intrahepatic cholesterol to bile acids, adsorbing cholesterol, or impeding cholesterol emulsification by bile, as well as delaying the diffusion of cholesterol in the intestinal lumen to intestinal epithelial cells. Dietary fiber and metabolites of intestinal microbiota can selectively alter the composition of intestinal flora, which in turn increases intestinal short-chain fatty acid (SCFA) levels, decreases the expression levels of inflammatory factors, and influences the expression of hunger fasting-inducing factors, thus improving the imbalance of glucose–lipid metabolism in the body [5,6,7].

Purple-grain wheat is a special type of wheat expressing blue, purple, or a mixture of both colors. Long-term consumption of purple-grain wheat flour products can improve immunity, constipation, calcium deficiency, hypertension, high blood pressure, high blood cholesterol, coronary heart disease, and diabetes, with a certain therapeutic effect on a variety of tumors and other diseases [8]. For example, purple-grain wheat Jizimai No. 14 (PWP) exhibits rich nutritional value. Specifically, the contents of the trace elements zinc, iron, magnesium, calcium, and selenium are 4.1, 2.6, 2.5, 1.6, and 1.5 times higher than those of common wheat, respectively; it also contains essential amino acids such as lysine, leucine, alanine, and histidine, and the dietary fiber content is 144 g/kg, which is 18 times higher than that of common wheat [9]. Previous studies have found that whole purple-grain wheat flour could significantly increase serum high-density lipoprotein cholesterol (HDL-C) and reduce serum total cholesterol (TC), triglyceride (TG), and low-density lipoprotein cholesterol (LDL-C) levels in hyperlipidemic rats. It was also found to effectively regulate lipase metabolism, enhance the active substances of vascular endothelial cells, and prevent the exacerbation of hepatic tissue damage due to abnormalities in lipid metabolism; thus, whole purple-grain wheat has the effect of improving lipid metabolism disorders [10]. Moreover, clinical trials have found that replacing part of the staple diet with purple-grain wheat improved glycemic and inflammatory distributions in patients with type 2 diabetes mellitus, as evidenced primarily by significant reductions in glycated albumin and interleukin (IL)-6 levels [11].

In this study, we propose to use Jizimai No. 14, a specialty purple-grain wheat from the Hebei Academy of Agriculture and Forestry, to study the effect of its action as a prebiotic, and to conduct dietary intervention tests at different doses on diet-induced hyperlipidemia mice and type 2 diabetic mice to investigate the regulation of glucolipid metabolism in hyperlipidemia and type 2 diabetes mellitus by purple-grain wheat Jizimai No. 14. This study will reveal the effects of purple-grain wheat on body weight, lipid metabolism, and glucose metabolism in mice, providing a theoretical basis for the dietary alleviation of metabolic diseases such as hyperlipidemia and type 2 diabetes mellitus and laying the groundwork for the development and utilization of lipid-lowering and glucose-lowering products made from specialty purple-grain wheat.

## 2. Materials and Methods

### 2.1. Agricultural Paste/Juice Preparation

Appropriate amounts of purple-grain wheat (Jizimai No. 14, from the Hebei Academy of Agriculture and Forestry, Shijiazhuang, China), cucumber seeds (*Cucumis satiuus* L., purchased from Hebei Kangpai Chinese Herbal Medicine Co., Ltd., Baoding, China), asparagus (*Asparagus officinalis* L., purchased from Qinhuangdao Changsheng Agricultural Technology Development Co., Ltd., Qinhuangdao, China), and carrots (*Daucus carota* var. *sativa* Hoffm., planted in the experimental plot of Hebei Academy of Agriculture and Forestry Sciences, Shijiazhuang, China) were placed in the tissue grinder (Ningbo Xinzhi Biotechnology Co., Ltd., Ningbo, China); the mass ratio to deionized water was 1:1. The combination of purple-grain wheat and cucumber seeds was chosen as the nourishing paste option, and that of asparagus and carrots was chosen as the juice option. After the cell wall-breaking was completed, the samples were filtered with 100-mesh gauze to obtain more refined forms of purple-grain wheat paste (PWP), cucumber seed paste (CSP), asparagus juice (AJ), and carrot juice (CJ), respectively, and the above pastes/juices were sterilized at 115 °C for 30 min and stored in the refrigerator at 4 °C until use.

### 2.2. Bacteria and Culture

*Bifidobacterium longum* subsp. *longum* NCTC11818 and *Lactobacillus acidophilus* NCFM were obtained from the American Type Culture Collection (ATCC) (Rockville, MD, USA). *Lacticaseibacillus paracasei* JY062 [12] and *Lacticaseibacillus rhamnosus* JL-1 [13] were isolated from traditional fermented milk from Tibet and healthy infant feces, respectively. *B. longum* NCTC11818 was cultured in modified De Man, Rogosa, and Sharpe (MRS) broth (Qingdao Hope Bio-Technology Co., Ltd., Qingdao, China) at 37 °C under anaerobic conditions for 24 h. *L. acidophilus* NCFM, *L. paracasei* JY062, and *L. rhamnosus* JL-1 were cultured in MRS broth (Qingdao Hope Bio-Technology Co., Ltd., Qingdao, China) at 37 °C under anaerobic conditions for 20 h to obtain activated suspensions.

### 2.3. Viable Bacteria Count

The prepared PWP, CSP, AJ, and CJ were added to the (modified) MRS broth medium according to the concentration gradient of 0%, 2%, 4%, and 8% *v*/*v*, respectively. Each group was inoculated with probiotics according to a 1% inoculum and incubated at 37 °C for 12 h for the viable bacteria count. Using the gradient dilution and decantation method, the diluted culture solutions were incubated in (modified) MRS agar at 37 °C under anaerobic conditions for 48 h. Three parallel experiments were performed, and the viable bacteria counts were recorded.

### 2.4. Hyperlipidemic Mouse Model and Experimental Design

SPF-grade male C57BL/6J mice (18 ± 2 g, 5 weeks old) were purchased from Vital River Laboratory Animal Technology Co., Ltd. (Beijing, China). Sixty male C57BL/6J mice were housed at a temperature of 22 ± 2 °C, a humidity of 55 ± 5%, and a 12 h day/night cycle. The mice were first acclimatized for 2 weeks on basal feed and ad libitum water. Subsequently, the mice were randomly divided into five groups (*n* = 12 per group), including the normal group (N), the high-fat diet group (HFD), the high-dose purple-grain wheat group (H), the medium-dose purple-grain wheat group (M), and the low-dose purple-grain wheat group (L). The mice in the N group were fed normal basal chow, and the mice in the other four groups were fed high-fat chow (60% kcal fat diets, 60% kcal Fat Diets 12492M, Beijing Bo’aigang Trade Center, Beijing, China) to induce the hyperlipidemic mouse model for 4 weeks. After that, mice in the H, M, and L groups were administered with 15 g/(kg·d), 7.5 g/(kg·d), and 3.75 g/(kg·d) of PWP by gavage for 4 weeks, while mice in N and M groups were received an equal amount of saline solution (0.85% w/v sodium chloride). The nutritional characteristics of purple-grain wheat (Jizimai No. 14, independently bred by the Hebei Academy of Agricultural and Forestry Sciences, Shijiazhuang, China) are shown in Table 1. The mice were fasted for 12 h and anesthetized by intraperitoneal injection of ketamine and diazepam before sacrificing at the end of the experiment.

#### 2.4.1. Body Weight

The body weights of the mice were measured and recorded weekly from the first week until the end of the experiment.

#### 2.4.2. Physiological Indicators

After 4 weeks of continuous dietary intervention, blood was removed from the eyeballs, left to stand for 1 h at 37 °C, centrifuged at 3000 rpm/min for 10 min, and the supernatant was removed to determine the levels of serum TC, TG, HDL-C, and LDL-C using the TC kit, TG kit, HDL-C kit, and LDL-C kit (Shanghai Enzyme-Link Biological Co., Ltd., Shanghai, China).

### 2.5. Type 2 Diabetes Mouse Model and Experimental Design

SPF-grade male C57BL/6J mice (18 ± 2 g, 5 weeks old) were purchased from Vital River Laboratory Animal Technology Co., Ltd. (Beijing, China). Sixty male C57BL/6J mice were housed at a temperature of 22 ± 2 °C, a humidity of 55 ± 5%, and a 12 h day/night cycle. The mice were first acclimatized for 2 weeks on basal feed and ad libitum water. Subsequently, the mice were randomly divided into five groups (*n* = 12 per group), including a negative control group (NC), a diabetes model group (DM), a high-dose purple-grain wheat group (HB), a medium-dose purple-grain wheat group (MB), and a low-dose purple-grain wheat group (LB). The mice in the NC group were fed normal basal chow, and the mice in the other four groups were fed high-fat and high-sugar chow (35% kcal high-fat feed, specifically 66.5% basal, 10% lard, 20% sucrose, 2.5% cholesterol, and 1% sodium cholate, Beijing Bo’aigang Trade Center, Beijing, China). At the end of the 4th and 5th weeks of the test cycle, mice in the modeling groups were injected intraperitoneally with 100 mg/kg of streptozotocin (STZ) solution (dissolved in 0.1 mol/L citrate buffer), and mice in the NC group were injected with an equal amount of citrate buffer, respectively. After that, mice in the HB, MB, and LB groups were administered with 15 g/(kg·d), 7.5 g/(kg·d), and 3.75 g/(kg·d) of PWP via the gavage method, and mice in NC and DM groups received an equal amount of saline solution (0.85% w/v sodium chloride) for 5 weeks. Then, the mice were fasted for 12 h and anesthetized by intraperitoneal injection of ketamine and diazepam before sacrificing at the end of the experiment.

#### 2.5.1. Body Weight

The body weights and blood glucose values of the mice were measured and recorded weekly from the first week until the end of the experiment.

#### 2.5.2. Blood Glucose

After the mice were fasted overnight without food or water, blood was taken from their tails, and the fasting blood glucose (FBG) values were measured with a blood glucose meter.

### 2.6. Statistical Methods

The data were expressed as the mean ± standard deviation (mean ± SD) and analyzed using SPSS 25.0, and statistical analyses between groups were compared using one-way analysis of variance (ANOVA). Graphs were plotted using Origin Prob 2024 and GraphPad Prism 8.02, with *p* < 0.05 representing a significant difference.

## 3. Results

### 3.1. Effect of Agricultural Products on the Growth of Probiotics

**Figure 1:**
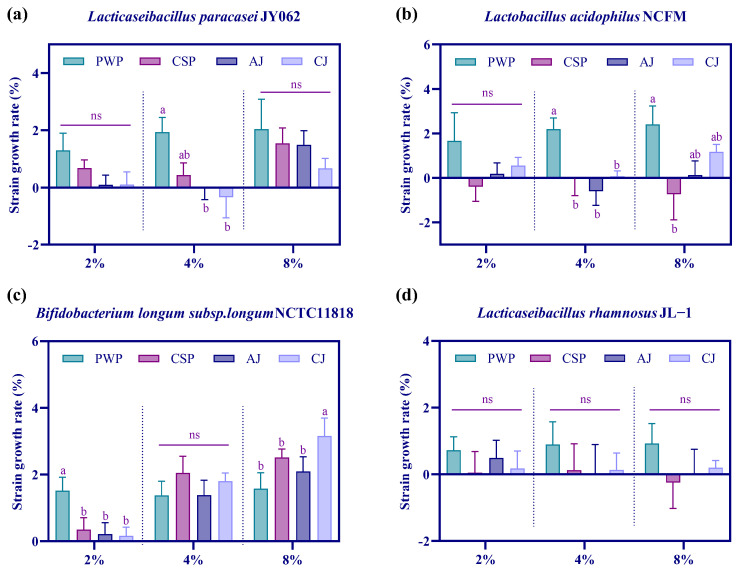
Effect of purple-grain wheat paste (PWP), cucumber seed paste (CSP), asparagus juice (AJ), and carrot juice (CJ) on the viable bacteria count of *Lacticaseibacillus paracasei* JY062 (**a**), *Lactobacillus acidophilus* NCFM (**b**), *Bifidobacterium longum* subsp. *longum* NCTC11818 (**c**), and *Lacticaseibacillus rhamnosus* JL-1 (**d**). Different superscripted letters in the same column indicate significant differences (*p* < 0.05).

The effect of PWP, CSP, AJ, and CJ on the growth of *L. paracasei* JY062, *L. acidophilus* NCFM, *B. longum* NCTC11818, and *L. rhamnosus* JL-1 was investigated, and the results are shown in Figure 1. In regards to the effect of viable bacteria on *L. paracasei* JY062, although there was no significant difference in the growth rate of *L. paracasei* JY062 at 2% and 8% product additions (*p* > 0.05), the growth rate of *L. paracasei* JY062 was higher than that for other agricultural products after PWP addition. And at 4% of the agricultural products added, the growth rate of *L. paracasei* JY062 after PWP addition was significantly higher than that of either AJ or CJ (*p* < 0.05) and higher than that of CSP. Among the effects of different agricultural products on the viable bacteria of *L. acidophilus* NCFM, there was no significant difference in the growth rate of *L. acidophilus* NCFM when the agricultural products were added at 2% (*p* > 0.05). At 4% agricultural product addition, the growth rate of *L. acidophilus* NCFM after PWP addition was significantly higher than that for other agricultural products (*p* < 0.05). At 8% agricultural product addition, the growth rate of *L. acidophilus* NCFM after PWP addition was significantly higher than that of CSP (*p* < 0.05). Overall, we found that PWP addition had the greatest effect on the growth of *L. acidophilus* NCFM. In addition, at 2% product addition, the growth rate of *B. longum* NCTC11818 was significantly higher that for the other products. There was no significant difference between the effects of the four agricultural products on the growth of *B. longum* NCTC11818 at 4% agricultural product addition (*p* > 0.05), while all four agricultural products promoted the growth of *B. longum* NCTC11818 at 8% addition, with CJ having the most significant effect (*p* < 0.05). We also explored the effect on the viable bacteria of *L. rhamnosus* JL-1 and found that there was no significant difference in the effect of 2%, 4%, and 8% additions on the growth of *L. rhamnosus* JL-1 (*p* > 0.05), but the maximum growth of *L. rhamnosus* JL-1 was observed in the presence of PWP. Therefore, it was found that PWP may play a prebiotic role and be effective in promoting the growth of probiotics (*L. paracasei* JY062, *L. acidophilus* NCFM, *B. longum* NCTC11818, and *L. rhamnosus* JL-1).

### 3.2. Effects of PWP on Body Weight in Hyperlipidemic Mice

**Figure 2:**
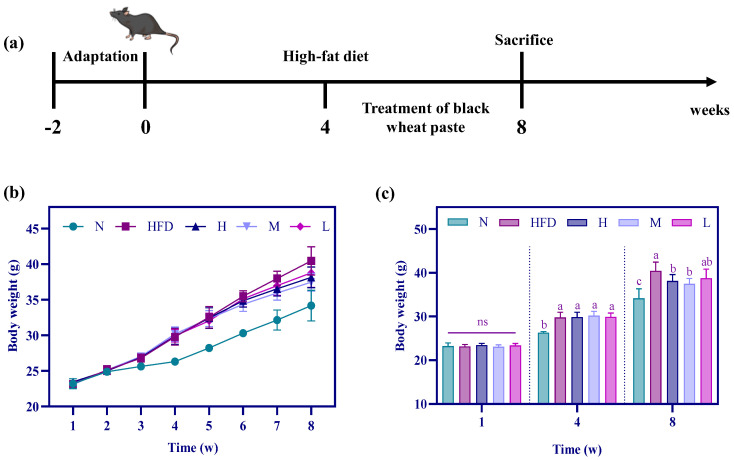
Simplified diagram of the hyperlipidemia model (**a**), mice weekly body weight curves (**b**), and the body weights of mice at the end of week 1, 4, and 8 (**c**). Different superscripted letters in the same column indicate significant differences (*p* < 0.05).

We established a hyperlipidemic mouse model via a high-fat diet, observed the mice’s status, and recorded their weight changes every week. The mice in the N group exhibited smooth hair, were agile, and showed healthy condition. The mice in the HFD group exhibited greasy hair and were slow. After the intervention of PWP, the mice’s hair returned to a soft state, the mice moved freely, and their condition was significantly improved. The body weights of the mice were determined at the end of each week, and the results are shown in Figure 2, indicating that the body weight of the mice consuming both normal and high-fat diets showed an upward trend over 8 weeks. In addition, there was no significant difference in the body weights of the mice in each group at the end of the first week, and it is possible that the change in body weight of the mice was not significant after consuming the high-fat diet for only 1 week (*p* > 0.05). Throughout the 4 weeks of consuming a high-fat diet, the mice in the HFD group showed a steady increase in body weight, which was 13.7% higher than that of the mice in the ND group (29.9 g vs. 26.3 g), indicating successful modeling of hyperlipidemic mice. Subsequently, by intervening with PWP for 4 consecutive weeks in mice in groups H, M, and L, we found that the body weight continued to increase as the mice grew, and at week 8, the body weight of mice in the HFD group was significantly increased compared to that of group N (*p* < 0.05). The weight gain due to the high-fat diet was reversed in the H, M, and L groups, where the mitigation of the body weight gain of the mice was more pronounced after the interventions of both the high and medium doses of PWP.

### 3.3. Effects of PWP on Blood Lipids in Hyperlipidemic Mice

When the body consumes a high-fat diet over a long period of time, this contributes to an accelerated rate of fat catabolism so that lipid synthesis is inhibited, resulting in the production of excess fatty acids and leading to elevated levels of TC and TG in the serum. Simultaneously, HDL-C is removed by lipase at an accelerated rate, which is accompanied by a decrease in HDL-C levels and an increase in LDL-C levels. An excess of TG in the body increases the weight of the adipose tissue and causes fatty liver; therefore, the more TG in the serum, the more serious the accumulation of fat in the mice. As shown in Table 2, the long-term high-fat diet significantly increased the serum levels of TC, TG, and LDL-C in mice, and the level of HDL-C decreased very significantly (*p* < 0.05). After 4 weeks of gavage with PWP, the serum levels of TC, TG, and LDL-C in mice showed a decreasing trend, and HDL-C showed an increasing trend. In the H group, mice showed a decrease in serum TC and an increase in serum HDL-C, with the TC content decreasing by 12.4% and the HDL-C content increasing by 17.5%, whereas, in the L group, the mice showed the greatest decrease in serum TG and LDL-C, with a decline of up to 34.2% and 38.8%, respectively. From the above, it was shown that gavage with PWP could improve the blood lipid level disorder induced by a high-fat diet in mice and further reduce the fat accumulation, to a certain extent.

### 3.4. Effects of PWP on Body Weight in Type 2 Diabetes Mice

**Figure 3:**
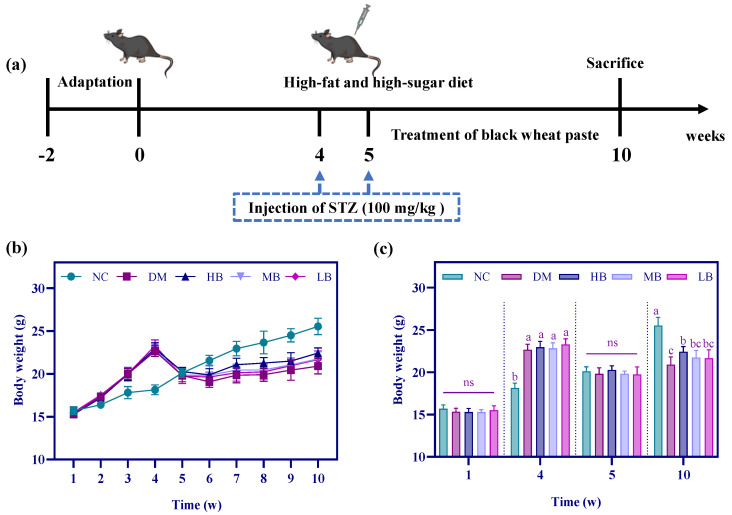
Simplified diagram of the type 2 diabetes model (**a**), mice weekly body weight curves (**b**), and the body weights of mice at the end of week 1, 4, 5, and 10 (**c**). Different superscripted letters in the same column indicate significant differences (*p* < 0.05).

We established a diabetic mouse model by STZ and determined the body weight of the mice at the end of each week; the results are shown in Figure 3. At the end of the 1st week, there was no significant difference in the body weight of the mice in each group, probably due to the insignificant change in body weight after consuming the high-fat and high-sugar chow for 1 week (*p* > 0.05). The body weight of the mice in each group increased continuously during the first 4 weeks, and at the end of the 4th week, the body weights of the mice in the DM, HB, MB, and LB groups that consumed the high-fat and high-sugar diets were significantly higher than those of the mice in the NC group (*p* < 0.05). At this time, the first STZ injection was administered to the mice, and it is worth noting that at the After 1 week of STZ injection, i.e., at the end of week 5, the weights of the mice in the DM, HB, MB, and LB groups decreased significantly (*p* < 0.05), while the weight of the mice in the NC group, which had not been injected with the STZ injection, did not show a decreasing trend, but rather a steady increase in weight. At this time, there was no significant difference in the body weights of the five groups of mice (*p* > 0.05). Simultaneously, at the end of the 5th week, mice in the DM, HB, MB, and LB groups consuming a high-fat, high-sugar diet were injected with a second dose of STZ. Subsequently, at the beginning of the 6th week, the mice in the HB, MB, and LB groups were intervened with high, medium, and low doses of PWP for 5 consecutive weeks, and we found that the mice continued to increase their body weights during this period. At the end of the 10th week, the body weights of mice in the DM group were significantly lower than those of the mice in the NC group (*p* < 0.05), whereas the body weights of mice intervened with PWP increased, and this was more pronounced in mice in the HB group, narrowing the gap between them and those in the normal group, suggesting that the body weight of the type 2 diabetes mice was improved by the intervention with PWP.

### 3.5. Effects of PWP on Blood Glucose in Type 2 Diabetes Mice

**Figure 4:**
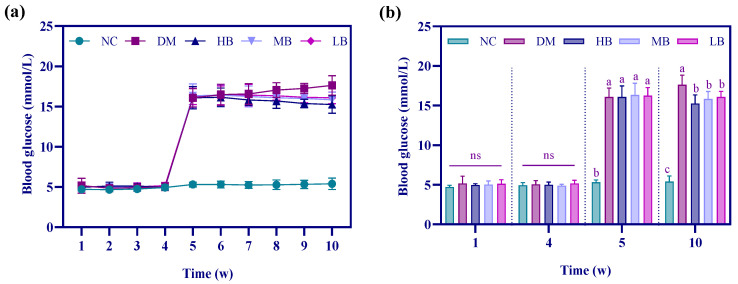
Mice weekly blood glucose curves (**a**) and the blood glucose of mice at the end of week 1, 4, 5, and 10 (**b**). Different superscripted letters in the same column indicate significant differences (*p* < 0.05).

We examined the FBG levels of the mice at the end of each week, and the results are shown in Figure 4. There was no significant change in the blood glucose of the mice in the first 4 weeks, indicating that the mice’s ability to regulate blood glucose was not affected by the high-fat and high-sugar diet after 4 weeks, and the level could be maintained at about 5 mmol/L in each group. At the end of the 4th week, the first dose of STZ was administered to the mice, and it notably produced a significant increase in FBG (*p* < 0.05) at the end of the 5th week in the DM, HB, MB, and LB mice, whereas the blood glucose of the mice in the NC group, which had not been injected with STZ, did not show any increasing trend, remaining at a stable equilibrium state. At the end of the 5th week, a second injection of STZ was administered to the mice in the DM, HB, MB, and LB groups consuming high-fat and high-sugar diets. Meanwhile, the intervention of PWP for 5 consecutive weeks was initiated in mice in the HB, MB, and LB groups, and we found that at the end of the 10th week, the blood glucose of mice in the DM group was still elevated, which indicated that the mice could be placed in a hyperglycemic state after the second injection of STZ. In contrast, PWP intervention significantly alleviated the hyperglycemic levels of the mice (*p* < 0.05). This suggests that PWP produces a statistically significant lowering effect on blood glucose compared with the levels in the untreated diabetic animals, but the reduction in glucose was small.

## 4. Discussion

Agricultural products are rich in nutrients that produce greater functional value for the human body. For example, carrots contain a large number of substances that are beneficial to the body’s health, such as carotenoids, fiber, and polyphenols [14]. Carrot supplementation has been shown to be associated with a lower body mass index (BMI) and alleviation of metabolic dysfunction [15]. Asparagus is high in nutritional value, low in calories, contains a variety of active ingredients, and exhibits cholesterol-lowering and hepatoprotective effects, as well as displaying anti-tumor and anti-diabetic properties [16]. Cucumber seeds are rich in potassium salt, vitamin A, vitamin E, and trace elements such as calcium, phosphorus, iron, etc., resulting in calcium supplementation; they are also rich in polysaccharides such as xylose and fructose, producing laxative effect and aiding in the intestinal treatment of constipation [17]. In this experiment, we chose PWP, CSP, AJ, and CJ to study the ability of probiotics to utilize them and to preliminarily determine their effects on probiotics. We found that PWP promoted the growth of *L. paracasei* JY062, *L. acidophilus* NCFM, *B. longum* NCTC11818, and *L. rhamnosus* JL-1, with the advantage of being a prebiotic. PWP, as one of the main components of human dietary grains, contains a large amount of dietary fiber, and purple-grain wheat Jizimai No. 14 offers a significant advantage in terms of dietary fiber content [9], a non-digestible food component with prebiotic potential that selectively stimulates the growth and/or activity of beneficial bacteria such as *Bifidobacteria* and *Lactobacillus* in the colon, thereby improving the health of the host [18].

Based on the results of the previous evaluation of the nutrient composition of Jizimai No. 14 purple-grain wheat, we found that as compared to common wheat, it is rich in functional components, including the trace minerals zinc, iron, magnesium, calcium, and selenium, as well as the free amino acids lysine, leucine, alanine, and histidine [9]. Therefore, based on the influence of dietary intervention on the intestinal microenvironment of mice, and the potential of Jizimai No. 14 purple-grain wheat as a prebiotic, as well as its compositional advantages, the present study was undertaken to explore its ability to regulate blood lipids in hyperlipidemic mice and blood glucose in mice with type 2 diabetes mellitus.

Food induction is commonly applied to establish hyperlipidemic mouse models, of which high-fat chow induction to establish hyperlipidemic mouse models is a more common modeling method in current studies [19,20]. Hu et al. established a mouse model of hyperlipidemia by feeding with a high-fat diet for 4 consecutive weeks, and at the end of the 4th week, the body weight, TC, TG, and LDL-C of the mice in the HFD group were all significantly higher than the levels of the mice in the N group, and the establishment of the hyperlipidemia model was determined to be successful [21]. In the present study, the same induction method (high-fat diet-fed mice) was used, and it was observed that the high-fat-diet-induced mice showed an increasing trend in body weight with the prolongation of feeding time.

The state of the mice was constantly observed during the test period, and it was found that the mice in the N group were active and exhibited smooth and silky fur, while the mice in the model group were inactive and displayed greasy and rough fur. After 4 weeks of gavage intervention, the state of the hyperlipidemic mice was significantly improved, which may be due to the regulation of blood lipid levels in mice after PWP intervention. With the prolongation of modeling time, the weight of mice still showed an increasing trend, but compared with the HFD group, the trend of weight increase in the mice after gavage with high, medium, and low doses of PWP was slow. The PWP intervention had an inhibitory effect on the weight gain of mice, indicating that the intervention with PWP could effectively inhibit the weight gain of hyperlipidemic mice. This may be related to the fact that the main component of PWP is starch, which at a higher intake, is not as effective in reducing weight. In brief, this was consistent with the results of Lan et al. [22], indicating that purple-grain wheat could inhibit the body weight of hyperlipidemia mice.

Hyperlipidemia is mainly characterized by dyslipidemia due to the imbalance of energy intake and consumption, accompanied by phenomena such as abnormal lipid metabolism, specifically manifested by elevated serum levels of TG, TC, and LDL-C, and decreased HDL-C levels [23]. In this study, the serum levels of TG, TC, and LDL-C of mice in the HFD group were significantly elevated, and the HDL-C level was significantly decreased. After gavage with different doses of PWP, the contents of TG, TC, and LDL-C in the serum of mice were reduced to different degrees, which caused the rate of HDL clearance by lipase to be inhibited and led to the increase in HDL-C content, indicating that PWP produced a better improvement effect on the lipid disorders induced by hyperlipidemia. The study of Junejo et al. was consistent with the results of the present study [24]. In addition, the high dietary fiber content of PWP reduces body weight, to a certain extent, in hyperlipidemic mice, and the hypolipidemic effect of wheat dietary fiber has been demonstrated [9,25]. Moreover, PWP is rich in mineral elements such as iron, calcium, magnesium, and zinc, which have a regulatory effect on lipid metabolism disorders [26,27].

T2DM is a chronic inflammatory disease characterized by insulin resistance and/or insufficient insulin secretion, mainly due to impaired pancreatic β-cell function. Long-term high-fat, high-sugar diets can induce insulin resistance, while STZ can specifically destroy pancreatic β-cells, so the combination of high-fat, high-sugar, and STZ has been widely used in mouse T2DM modeling to mimic the metabolic properties of human T2DM [28,29]. The experiment was conducted using STZ for modeling; specifically, at the end of the 4th and 5th weeks, STZ solution was injected intraperitoneally at 100 mg/kg, respectively, and FBG was used as an index to determine whether the modeling was successful or not, i.e., when the FBG exceeded 11.1 mmol/L, it indicated that the modeling was successful [30]. In this study, the FBG of all mice in the modeling group exceeded 11.1 mmol/L, indicating that T2DM modeling was successful.

The study also recorded the status of the mice after modeling and found that compared with the normal group, the model group mice appeared to be slow and insensitive to external stimuli. In the three dietary intervention groups, the above symptoms were improved to different degrees, along with the abnormal weight loss that is prone to occur in the development of T2DM. The body weight of the T2DM mice was significantly reduced after STZ injection (4–5 weeks of the test cycle), a phenomenon in which T2DM mice can only provide energy by burning proteins and fatty acids to meet their energy needs [31]. T2DM mice began to show a downward trend in body weight for 2 consecutive weeks, thus speculating that this phenomenon may be due to the longer time for the drug to take effect after two STZ injections, and as earlier studies have reported a decrease in body weight in diabetic animals, the phenomenon in the present study was consistent with the results of these earlier studies [32,33]. From the 7th week, the T2DM mice showed a persistent increase in body weight, and the trend of body weight increase in the HB group was significantly higher than that in the LB, MB, and DM groups. This suggested that PWP may be effective in ameliorating diabetic symptoms, to some extent, by consuming carbohydrates to meet energy requirements and inhibiting weight loss in diabetic mice.

Type 2 diabetes is a group of clinical syndromes characterized by chronic hyperglycemia [34]. In terms of the trend of FBG changes, the blood glucose values of the mice in the model group showed a significant increase both before and after the completion of modeling, and the blood glucose of the mice in the model group continued to increase with the extension of the time after modeling. In contrast, in all three PWP intervention groups, the FBG of mice gradually leveled off in the later stages of PWP intervention and showed a decreasing trend, with the best decrease in blood glucose of the mice in the HB group, which was similar to the results of the study by Tu et al. [35]. Earlier research demonstrated that zinc is an important component of insulin granules, which may regulate insulin secretion and signaling, and zinc supplementation could improve glucose homeostasis [36,37]. In this study, as zinc levels were significantly increased in PWP [9], it is hypothesized that PWP may be associated with this result.

Overall, PWP can regulate blood glucose and blood lipids, an effect which is closely related to its functional nutritional composition (high dietary fiber and mineral contents). Magnesium is a cofactor in more than 300 enzymatic reactions and is critical in the metabolism of adenosine triphosphate (ATP) in enzymatic reactions, as well as in regulating glucolipid metabolism [38]. Low dietary magnesium intake was found to be strongly associated with an increased risk of lipid metabolism disorders; however, an appropriate increase in magnesium within the appropriate intake may reduce the risk of type 2 diabetes [39]. Moreover, moderate iron supplementation alleviated high-fat-diet-induced weight gain and disturbed energy metabolism in mice by modulating the mitochondrial signaling pathways [27]. Several studies have shown that dietary fiber intake could alter glucose and lipid metabolism through microbial fermentation and the subsequent production of SCFAs, which could improve glucose and lipid parameters in individuals with metabolic dysregulation [40]. Therefore, PWP further illustrates its ability to regulate blood glucose and blood lipids from its compositional advantages.

## 5. Conclusions

In conclusion, PWP had a reducing effect on the body weight of mice in the hyperlipidemic model and was able to effectively reduce TC, TG, and LDL-C in the serum of hyperlipidemic mice and effectively increase HDL-C levels. In addition, PWP alleviated the weight loss and reversed the persistent hyperglycemia levels in type 2 diabetic mice and lessened the extent of diabetes. This is related to its nutritional components and its ability to act as a prebiotic. Therefore, PWP is a potential functional food for the regulation of glycolipid disorders.

## Figures and Tables

**Table 1:** The nutritional characteristics of Jizimai No. 14 purple-grain wheat [9].

Category	Ingredient	Content
Macronutrients (g/kg)	Protein	155
	Fat	19
	Starch	562
	Reducing sugar	14
	Dietary fiber	144
Minerals (mg/kg)	Zinc	29
	Iron	36
	Calcium	453
	Magnesium	1340
	Selenium	0.105
Essential amino acids (g/kg)	Lysine	4.7
	Threonine	3.3
	Methionine	3.2
	Phenylalanine	7.4
	Leucine	18.2
	Isoleucine	4.1
	Valine	11.1

**Table 2:** Effects of different treatment groups on blood lipids in mice using the hyperlipidemia model.

Groups	TC (mmol·L^−1^)	TG (mmol·L^−1^)	HDL-C (mmol·L^−1^)	LDL-C (mmol·L^−1^)
N	2.81 ± 0.17 a	1.14 ± 0.09 a	1.59 ± 0.14 a	1.01 ± 0.15 a
HFD	3.96 ± 0.79 b	1.93 ± 0.23 b	1.20 ± 0.11 b	2.37 ± 0.23 b
H	3.47 ± 0.19 b	1.27 ± 0.31 c	1.41 ± 0.09 c	1.67 ± 0.14 c
M	3.35 ± 0.45 b	1.31 ± 0.17 c	1.37 ± 0.07 c	1.51 ± 0.22 c
L	3.61 ± 0.27 b	1.27 ± 0.21 c	1.36 ± 0.11 c	1.45 ± 0.08 c

Note: Lowercase letters denote significance at the *p* < 0.05 level for the same indicator between different groups. Different letters in the same column indicate significant differences (*p* < 0.05).

## Data Availability

Data in the project are still being collected, but all data used in the study are available by contacting the authors.
